# 利用基因芯片技术分析胸腺瘤中基因的变异

**DOI:** 10.3779/j.issn.1009-3419.2020.102.46

**Published:** 2020-12-20

**Authors:** 立军 王, 磊 于, 鑫 杜, 承瑜 霍

**Affiliations:** 1 100123 北京，民航总医院 Civil Aviation General Hospital, Beijing 100123, China; 2 100069 北京，首都医科大学附属北京同仁医院胸外科 Department of Thoracic Surgery, Beijing Tongren Hospital, Capital Medical University, Beijing 100069, China

**Keywords:** 胸腺瘤, 基因芯片, 差异基因, 信号通路, Thymoma, Gene-chip, Abnormally expressed genes, Signaling pathways

## Abstract

**背景与目的:**

胸腺瘤是前纵隔最常见的恶性肿瘤，其具体的发病机制仍不十分清楚，这就限制了对胸腺瘤靶向药物的研究。本研究利用二代基因芯片技术分析胸腺瘤，了解胸腺瘤中表达显著变化的基因和信号通路，为胸腺瘤发病机制的研究提供帮助。

**方法:**

2015年1月-2017年12月，我们利用CapitalBio mRNA表达谱芯片技术分析了31例胸腺瘤，然后利用逆转录聚合酶链反应进行基因确证。

**结果:**

我们发现了一些胸腺瘤与瘤周围胸腺组织表达量有差异的基因的表达，其中6种在肿瘤基因组学中已证实的驱动基因（*FANCI*、*NCAPD3*、*NCAPG*、*OXCT1*、*EPHA1*和*MCM2*）在胸腺瘤中表达显著异常。对拷贝数变异所影响的具体基因进行检测，发现*E2F2*、*EPHA1*、*CCL25*和*MCM2*等上调明显的基因和*IL-6*、*CD36*、*FABP4*、*SH2D1A*、*MYOC*等下调明显的基因。利用KEGG数据库分析发现10条信号通路基因表达普遍上调或下调显著基因，如系统性红斑狼疮、病毒致癌基因、原发性免疫缺陷、细胞周期基因和p53信号通路等，可能与胸腺瘤的发生相关。

**结论:**

我们在胸腺瘤中发现了多种异常表达的基因，这将对今后胸腺瘤发病机理和生物标记物的研究提供参考。

## Abstract (en)

**Background and objective:**

Thymoma is the most common malignant tumor in anterior mediastinum, and its specific pathogenesis is still unclear. This limits the study of targeted drugs for thymoma. The aim of the study is to investigate the genes and signal pathways of thymoma, and provide help for the research of thymic tumor pathogenesis using the technology of second-generation genechip to analyze thymoma.

**Methods:**

From January 2015 to December 2017, we analyzed 31 cases of thymoma by CapitaBio mRNA expression profile genechip technology, and then confirmed the genes by reverse transcription-polymerase chain reaction (RT-PCR).

**Results:**

We found some genes with different expression levels between thymoma and surrounding thymus tissue. Among them, six driving genes (*FANCI*, *CAPD3*, *NCAPG*, *OXCT1*, *EPHA1* and *MCM2*) were significantly abnormal in thymoma. Some specific genes affected by copy-number variation were detected: *E2F2*, *EphA1*, *CCL25* and *MCM2* were significantly up-regulated, while *IL-6*, *CD36*, *FABP4*, *SH2D1A* and *MYOC* genes were significantly down-regulated. KEGG database analysis showed that the expression of 10 signaling pathway genes was generally up-regulated or down-regulated, such as systemic lupus erythematosus, viral oncogenes, primary immunodeficiency, cell cycle genes and p53 signaling pathway, which may be related to occurrence of thymoma.

**Conclusion:**

We found a variety of genes abnormally expressed in thymoma, which will provide reference for the study of pathogenesis and biomarkers of thymoma in the future.

胸腺肿瘤是一种来源于胸腺上皮细胞的最常见的前上纵隔肿瘤。胸腺肿瘤发病率较低，每年世界范围内每100, 000人仅发生0.32例^[1]^。胸腺肿瘤生长缓慢，组织成分繁多且无统一的组织学分类，可具有不同的生物学特性。这些生物学特性对胸腺肿瘤临床特点及疾病转归具有一定影响。

根据2004年世界卫生组织（World Health Organization, WHO）的分类，胸腺肿瘤分为胸腺癌和胸腺瘤，后者根据其组织学特征进一步分为A型、AB型、B1型、B2型和B3型^[2]^。目前认为所有的胸腺瘤均是潜在恶性的。我们以往的研究与国际上多项研究结果一致，认为WHO的胸腺瘤分型是独立的预后因素。A型和AB型胸腺瘤预后最好^[3-5]^，10年生存率接近100%，而侵袭性B2型和B3型胸腺瘤的10年生存率在70%左右。胸腺瘤常伴有副瘤综合征，如甲状腺功能亢进、单纯红细胞再生障碍性贫血、重症肌无力和内分泌病等，其中以重症肌无力最为常见^[6]^。国内外资料显示重症肌无力合并胸腺瘤发病率基本在10%-30%之间，而胸腺瘤合并重症肌无力在15%-60%之间。合并有重症肌无力的胸腺瘤与单纯胸腺瘤在临床病理特点上存在一定差别。

手术是治疗胸腺瘤的主要手段，预后主要由诊断时分期和肿瘤切除的完整性决定^[7]^。对不可切除和转移的胸腺瘤，化疗是主要治疗手段，进展期胸腺瘤对于全身化疗和局部放射治疗效果欠佳^[8]^，在我们的一项胸腺瘤化疗敏感程度评价的研究^[9]^中发现，在目前国内外临床研究中报道的11种对胸腺瘤可能有效的化疗药中，尽管胸腺瘤对表阿霉素的敏感性最高，也仅为16%，而胸腺瘤对力比泰的敏感性最低。由于对胸腺瘤基因表征异常的研究有限，胸腺瘤的靶向治疗研究进展受到阻碍。对不可切除和转移的胸腺瘤患者来讲，几乎无其他有效的治疗方法。因此，为改善胸腺瘤患者灰色预后现状，当前迫切需要对胸腺瘤发病机制进行深入研究。

## 1 资料与方法

### 1.1 一般资料

2015年1月-2017年12月，我们利用CapitalBio mRNA表达谱芯片技术分析了31例胸腺瘤的资料。患者男17例，女14例; 年龄27岁-74岁（中位年龄60岁）。经病理证实[根据WHO 2004版组织分型]：5例为AB型胸腺瘤，6例为B1型，12例为B2型，5例为B2型/B3型，3例为B3型; 单纯胸腺瘤6例，合并重症肌无力25例。按改良Masaoka临床分期：Ⅰ期2例，Ⅱ期18例，Ⅲ期9例，Ⅳ期2例。

### 1.2 方法

#### 1.2.1 标本采集

胸腺瘤和瘤旁胸腺组织标本采集于北京同仁医院手术过程中。收集的标本立即放在液氮中速冻，然后放入-80 ℃的冰箱中保存。

#### 1.2.2 CapitalBio mRNA表达谱芯片

用包含34, 000个人类mRNA探针的微阵列芯片（博奥生物股份有限公司，中国）对31份经手术切除的胸腺瘤和瘤旁胸腺组织进行分析。

### 1.3 RNA提取、标记和杂交

采用Trizol试剂（Invitrogen）从胸腺瘤和瘤周围胸腺组织中提取含有小RNA的总RNA，并参照试剂说明书使用mirVana miRNA分离试剂盒（Ambion, Austin, TX, USA）对总RNA进行纯化。使用分光光度计（NanoDrop ND-1000）对纯化后的总RNA进行定量，通过OD_260/280_读数判断RNA的纯度和浓度。用RNA 6000纳米芯片实验室试剂盒和生物分析仪2100（安吉伦技术公司，美国加州圣克拉拉）通过毛细管电泳检测RNA完整性。对RNA完整性值> 6的RNA样本进行分析。

### 1.4 微阵列成像与数据分析

利用GeneSpring V13.0软件（Agilent）分析lncRNA+mRNA阵列数据，进行数据汇总、标准化和质量控制。为了选择差异表达的基因，设置变化倍数≥2和≤-2及*t*检验*P*值为0.05作为筛选阈值。利用CLUSTER 3.0软件的调整数据功能，对数据进行Log2转换，以基因为中心，用平均连锁的层次聚类法进一步分析。最后，使用javatreeview（美国加州斯坦福大学医学院）进行树可视化。

### 1.5 RNA分离、cDNA合成及定量PCR（quantitative polymerase chain reaction, qPCR）

组织样本用功率均质器在1 mL的Trizol试剂（Invitrogen）中均质。然后根据制造商的说明从所有样品中分离总RNA。互补DNA（complementary DNA, cDNA）使用MMLV逆转录酶cDNA试剂盒（TAKARA）从1 μg总RNA中合成的。

设计了跨越内含子边界的qPCR反应引物，并用青科生物技术进行了合成。在Bio-RadCFX96实时PCR检测系统（Bio-RadCFX96 real-time PCR detection system, Bio-RadCFX96）上用SYBR Premix Ex-Taq（TAKARA）在96孔板上进行qPCR。用GAPDH引物作为内对照。比较Ct（ΔΔCt）法用于数据归一化。

### 1.6 逆转录聚合酶链反应（reverse transcription-PCR, RT-PCR）

取储存-80 ℃冰箱中的组织样本（胸腺瘤和瘤周围胸腺组织）38对，天平称重50 mg置于研钵中，加入液氮用研杵充分研磨。利用RT-PCR技术检测细胞中基因表达水平，对基因芯片中发现的显著差异表达的基因进行验证。

### 1.7 统计学方法

用GraphPad Prism 5.0进行统计分析，所有数据均采用均数±标准差（Mean±SD）表示。实验组间比较采用不配对*t*检验。*P* < 0.05为差异有统计学意义。

## 2 结果

### 2.1 表达量有差异的基因

初步发现了一些胸腺瘤与瘤周围胸腺组织表达量有差异的基因的表达，其中上调超过2倍的基因共有292个，超过5倍的基因有2个，差异基因表达上调前10位基因分别是：趋化因子*CCL25*、*HIST1H1B*、*SH2D1A*、*DNTT*、*PASK*、*CENPF*、*HIST1H2BD*、*S100A14*和*NPTX1*和*septin 6*等基因，上调显著基因主要集中在第19、第1、第6、第17和X染色体上; 下调超过2倍的基因共有596个，超过5倍的基因有115个，超过10倍的基因有21个，超过20倍的基因有6个，差异基因表达下调前10位基因分别是：*PLIN1*、*MYOC*、*ADH1A*、*FABP4*、*ADIPOQ*、*ADH1C*、*MGST1*、*LPL*、*CIDEC*和*CIDEC*等基因，下调显著基因主要集中在第15、第1、第4、第8和第3染色体上。对比已报道的众多关键癌基因，在上调超过2倍的基因中，本研究包括有*FANCI*、*NCAPD3*、*NCAPG*、*OXCT1*、*EPHA1*和*MCM2*等关键癌基因。接下来，在38例胸腺瘤标本中，通过RT-PCR对表达量有显著差异的基因进行验证，上调明显的基因包括*E2F2*、*EPHA1*、*CCL25*和*MCM2*等; 下调明显的基因包括*IL-6*、*CD36*、*FABP4*、*SH2D1A*和*MYOC*等（图 1）。

**图 1:**
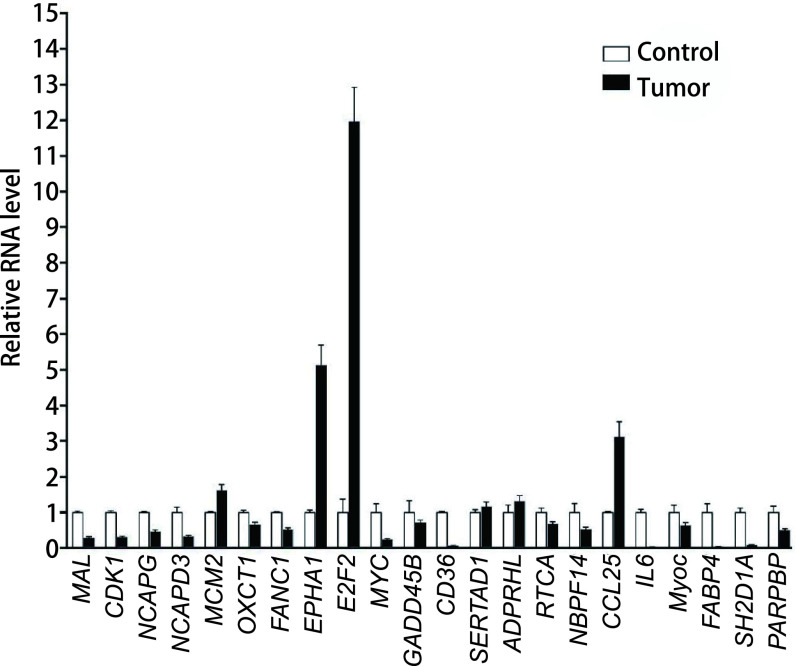
经RT-PCR确认，38例胸腺瘤样本中升高或下降显著的基因 Further confirmed by RT-PCR, the genes were expressed differentially.

RT-PCR: reverse transcription-polymerase chain reaction.

### 2.2 聚类分析

#### 2.2.1 非监督聚类分析

非监督聚类分析目的是通过对10, 000个基因分析基因表达差异，使差异基因表达相近的标本聚集在一起（图 2）。4种不同的集群分别有6个、3个、18个和4个肿瘤。仅集群2由特定的AB型肿瘤组成，群集1的6个肿瘤中，1个AB型肿瘤、1个B1型、2个B2型、1个B2型/B3型和1个B3型; 集群3中有1个AB型肿瘤，5个B1型肿瘤，8个B2肿瘤、2个B2型/B3型肿瘤和2个B3型肿瘤; 集群4中的4个肿瘤是由1个B2型肿瘤和3个B2型/B3型肿瘤组成。

**图 2:**
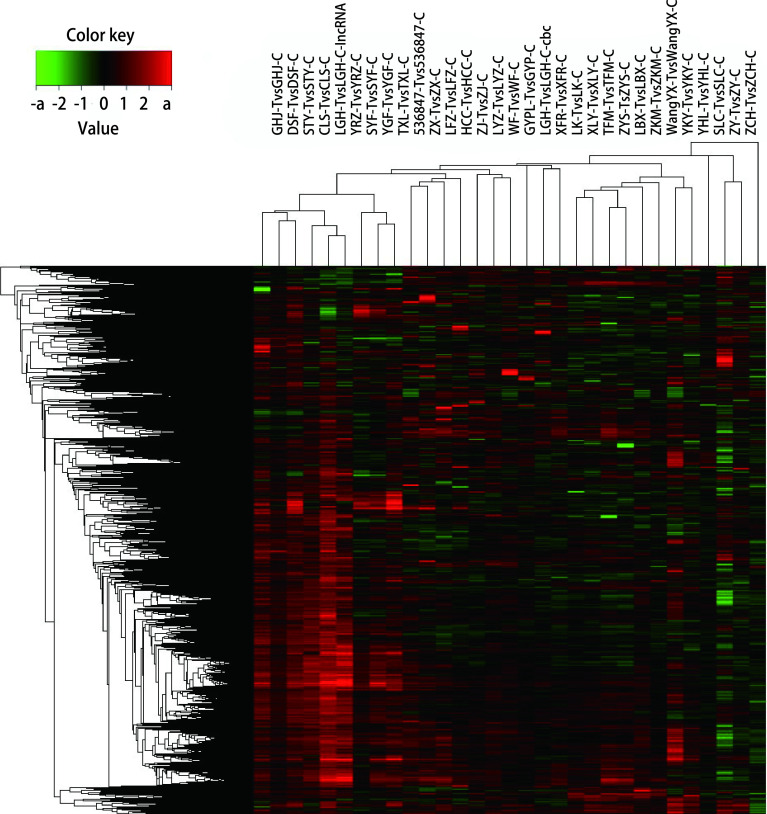
非监督聚类分析 The unsupervised cluster analysis

#### 2.2.2 监督聚类分析

在基因差异表达的基础上，选取上调2倍以上的基因和下调2倍以上的基因产生一个监督聚类热度图（图 3）。6种不同的集群分别有2个、8个、7个、7个、4个和3个肿瘤。集群1由特定的2个B2型肿瘤组成，集群6由特定的3个B2/B3型肿瘤组成。群集2的8个肿瘤中4个AB型肿瘤、1个B1型、1个B2型、1个B2型/B3型和1个B3型; 集群3中有3个B1型肿瘤，3个B2肿瘤和1个B2型/B3型肿瘤; 集群4中的7个肿瘤是由1个AB型肿瘤、1个B1型、3个B2型肿瘤、1个B2型/B3型肿瘤和1个B3型肿瘤组成; 集群5中有1个B1型肿瘤、1个B2肿瘤和1个B3型肿瘤。

**图 3:**
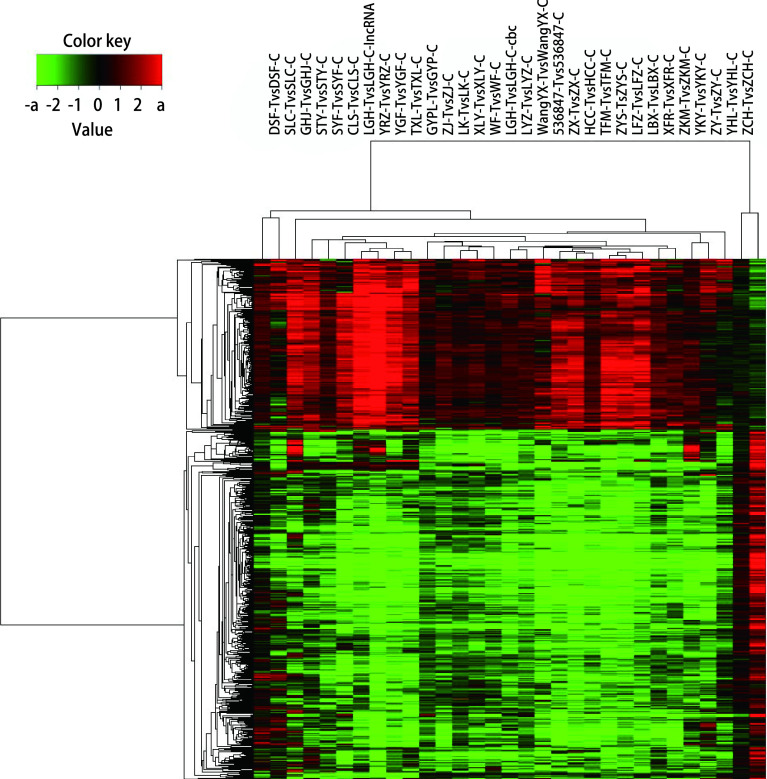
监督聚类分析 The supervised clustering heat map

### 2.3 表达异常的信号通路

利用生物信息学研究KEGG数据库分析发现，10条信号通路基因表达普遍上调或下调显著基因可能与胸腺瘤发生有相关性（图 4），即系统性红斑狼疮、病毒致癌基因、补体和凝血级联反应基因、造血细胞谱系基因、原发性免疫缺陷、细胞周期基因、ECM受体相互作用基因、PPAR信号通路、p53信号通路、癌症转录失调等信号通路。另外，我们发现以下4种疾病信号传导通路变化最显著，分别是单纯疱疹、原发性免疫缺陷、先天性全身性脂肪营养不良、经典补体途径成分缺陷等（图 5）。

**图 4:**
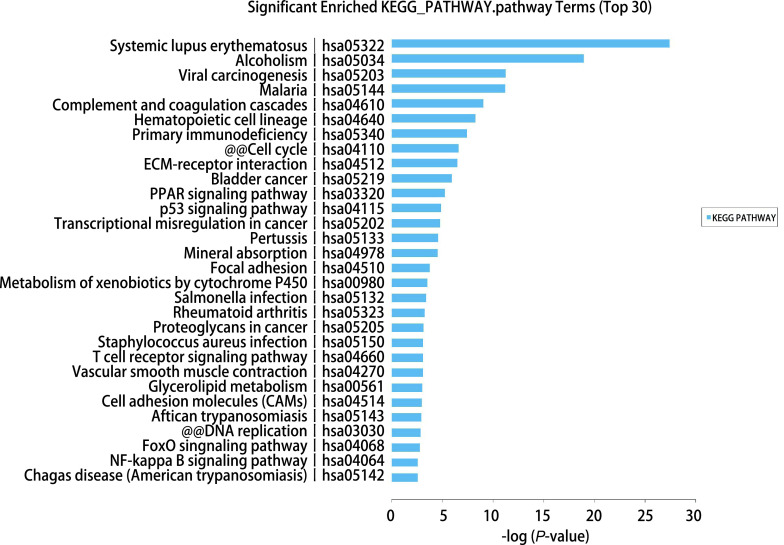
KEGG数据库分析发现有10条信号通路基因表达普遍上调2倍以上 KEGG database analysis suggested that ten top signaling pathways were identified in genes upregulated for more than 2-fold in thymoma

**图 5:**
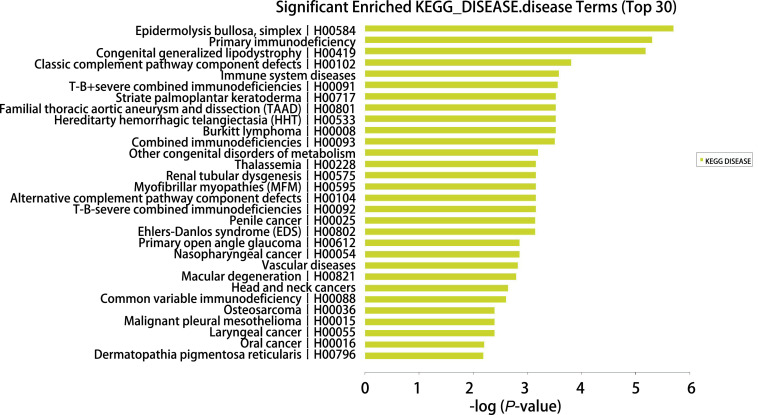
KEGG数据库分析发现的变化显著的疾病信号传导通路 KEGG database analysis found significant changes in disease signaling pathways

## 3 讨论

胸腺瘤具体的发病机制仍不十分清楚。人类肿瘤的发生是多种基因共同作用的结果。癌基因的激活和抑癌基因的失活或突变是其发生、浸润和转移的主要分子基础，胸腺瘤的发生亦不例外^[10, 11]^。

目前为止，得到国际普遍认同的是美国国家癌症研究所的学者们发现的A型胸腺瘤中*GTF2I*这一基因在胸腺瘤中的突变^[12]^。几乎所有的相对惰性胸腺瘤（生长缓慢，不具侵袭性）都带有*GTF2I*基因突变，但在恶性程度相对较高的B型胸腺瘤中，*GTF2I*基因突变较少见。尽管国内外报道^[13-16]^显示，胸腺肿瘤的发生、发展过程中有可能涉及到*CDKN2A*、*Galectin*、*c-myc*、T细胞受体β和γ基因和*SOX2*基因等多种癌基因的改变。但由于缺少胸腺瘤细胞株，研究者很难对这些基因可能的致胸腺瘤机制进行深入研究。

自2015年我们利用具有更高精度的全外显子和全基因组测序二代测序方法分析了31例胸腺肿瘤，检测到大量胸腺瘤新出现的基因的表达和胸腺瘤与瘤周围胸腺组织表达量有差异的基因的表达，并发现6种在肿瘤基因组学中已证实的驱动基因（*FANCI*、*NCAPD3*、*NCAPG*、*OXCT1*、*EPHA1*和*MCM2*）在胸腺瘤中表达显著异常。对拷贝数变异所影响的具体基因进行检测，研究发现*E2F2*、*EPHA1*、*CCL25*和*MCM2*等上调明显的基因和*IL-6*、*CD36*、*FABP4*、*SH2D1A*、*MYOC*等下调明显的基因。尤其是利用KEGG数据库分析发现的基因表达普遍上调的信号通路，如系统性红斑狼疮、病毒致癌基因、原发性免疫缺陷、细胞周期基因和p53信号通路等，这与国际上一些专家的研究相吻合。在胸腺瘤致病机制上，有学者^[17]^认为，某些病毒直接感染胸腺后通过基因分子水平的调控而致瘤，但一直以来，均未在胸腺和胸腺瘤内找到明确的病毒。通过我们的研究将为病毒感染致胸腺瘤的研究提供一定线索。另外，近几年，有学者^[18]^发现在某些胸腺瘤中存在*p53*基因突变，但由于缺少胸腺瘤细胞株，亦未能对其信号通路进行研究。

目前，国际上根据组织学特征对胸腺瘤进行分型，对胸腺瘤恶性程度评估和治疗具有一定临床意义。但很多胸腺瘤尽管组织分型相同，生物学特征却大相径庭。为了更为准确地判断胸腺瘤的生物学特征，我们尝试通过聚类分析方法对胸腺瘤进行分类。无论非监督聚类分析，还是监督聚类分析，我们均没能看到哪一集群与组织分型具有明显的一致性。

综上所述，我们利用二代基因测序技术发现了胸腺瘤中多种异常表达的基因，这将对今后胸腺瘤发病机理和生物标记物的研究提供参考。由于缺乏细胞株和动物模型，我们所检测到的异常表达的基因在胸腺瘤中的作用机制尚不能进一步证实。今后将利用我们培养的胸腺瘤细胞系对各基因致病的具体信号通路进行更为深入研究。
